# Automatic Rule Generation for Decision-Making in Context-Aware Systems Using Machine Learning

**DOI:** 10.1155/2022/5202537

**Published:** 2022-05-06

**Authors:** Roua Jabla, Maha Khemaja, Félix Buendia, Sami Faiz

**Affiliations:** ^1^Department of Computer Engineering, Universitat Politècnica Valencia, Camino de Vera S/N, Valencia 46022, Spain; ^2^ISITCom, University of Sousse, Sousse 4011, Tunisia; ^3^PRINCE Research Lab, ISITCom, University of Sousse, Sousse 4011, Tunisia; ^4^LTSIRS Laboratory, University of Tunis El Manar, Tunis 5020, Tunisia

## Abstract

With the increasing interest devoted to dynamic environments, a crucial aspect is revealed in context-aware systems to deal with the rapid changes occurring in users' surrounding environments at runtime. However, most context-aware systems with predefined context-aware rules may not support effective decision-making in dynamic environments. These context-aware rules, which take into account different context information to reach an appropriate decision, could lose their efficiency at runtime. Therefore, a growing need is emerging to address the decision-making issue leveraged by dynamic environments. To tackle this issue, we present an approach that relies on improving decision-making in the wake of dynamic environments through automatically enriching a rule knowledge base with new context-aware rules discovered at runtime. The major features of the presented approach are as follows: (i) a hybridization of two machine learning algorithms for rule generation, (ii) an extended genetic algorithm (GA) for rule optimization, and (iii) a rule transformation for the knowledge base enrichment in an automated manner. Furthermore, extensive experiments on different datasets are performed to assess the effectiveness of the presented approach. The obtained experimental results depict that this approach exhibits better effectiveness compared to some algorithms and state-of-the-art works.

## 1. Introduction

In context-aware computing, there exists a shift from static environments to dynamic environments [1]. This shift reflects a growing interest devoted to dynamic environments. With the growing interest, a crucial need is revealed for context-aware systems to be aware of and to adapt to their changing contexts in highly dynamic environments at runtime [2]. To support this need, a grand challenge is that context-aware systems should adjust their behaviors to the dynamics entailed in their surrounding environments at runtime. In order to meet this challenge, a decision-making process improvement by providing appropriate services to users situated in highly changing environments has emerged to make context-aware systems more resilient to dynamic environments at runtime. Nevertheless, at present, most context-aware systems usually work well in static environments with predefined context-aware rules that can only behave to changes in environment attributes and context information [3]. They can handle neither dynamic environments nor context changes at runtime since predefined rules might not be suitable for the dynamic nature of environments. On that account, rules need to be constantly evolved to remain relevant over time [4]. This raises more attention to be paid to how to enrich a rule knowledge base through the generation of efficient rules to timely react to arising environment changes at runtime. As data mining and specifically machine learning could be more accurate for profiting better rules [5], exploring recent advances in the application of machine learning might be beneficial in offering a way to learn and generate a set of nonredundant rules that are easily comprehensible and are capable of representing contextual knowledge in a very clear and efficient way.

To achieve this sustained attention, we propose, in this work, an approach that aims to automatically improve the decision-making process to support dynamic environments at runtime. The main feature of the proposed approach is to offer a rule knowledge base, where context-aware rules are fluid and evolutive at runtime for alleviating the burden of manually creating rules to react toward users' environment changes. The novelty and contribution of this approach could be drawn from threefold: first, we present a novel hybrid learning approach toward effectively generating a concise set of nonredundant association rules in an automated fashion by applying two learning algorithms. We hybridize machine learning algorithms to generate a more accurate and complete set of rules, to avoid redundancy, and to build a strong rule knowledge base in a context-aware system, in which we are interested. Second, we extend a genetic algorithm (GA) [6] with a multianalysis technique in the direction of rule optimization. The rule optimization is applied to find the well-performed rules from the generated rules since machine learning algorithms are not much proficient at optimizing rules. Third, we introduce an automatic transformation of obtained association rules to rules expressed in Jena [7]. Rule transformation is performed to express the well-performed rules in such a way that they can run over an ontology-based context model and a context-aware system can reason. Moreover, we conduct a range of experiments to assess the effectiveness of the proposed approach on different datasets from the UCI Machine Learning Repository [8]. First, we compare the proposed approach with the traditional association rule mining algorithms to analyze the number of generated rules. Then, we evaluate the performance of the proposed approach with the most common machine learning algorithms and certain state-of-the-art works. Finally, we analyze the computational time of the proposed approach and the machine learning algorithms. The provided results prove the effectiveness of the proposed approach by achieving the best result in terms of the number of rules, precision, recall, and accuracy among certain state-of-the-art works and algorithms such as apriori, FP growth, K-nearest neighbor, Naïve Bayes, JRip, and decision table.

The rest of this study is arranged as follows. We review the related work in Section 2. In Section 3, we introduce the proposed approach and outline the overall architecture and modules in detail. We present the experimental setup and the results obtained with an example of generated context-aware rules in Section 4. In Section 5, we illustrate the discussion of the results. Finally, we draw conclusions and highlight the direction for future work in Section 6.

## 2. Related Work

Data mining, especially machine learning and association rule mining, algorithms play a vital role in rule discovery from data. By searching through the literature, there is an extensive research basis to support association rule discovery from data to tackle the challenge of the decision-making improvement. In the following, we review first some works for discovering rules within the area of context awareness. Then, we provide a discussion to highlight the research gaps that motivate us to propose our approach.

A large number of works for association rule learning have been introduced using association rule mining algorithms. In this sense, Gabroveanu and Diaconescu [9] proposed a recommender system for students. They used data obtained from the learning database and apriori as an association rule mining algorithm in order to identify strong association rules for students. Then, these rules, obtained in an offline mining process, are translated into Jena rules to allow reasoning over RDF models. In addition, Kaliappan and Sai [10] presented a new modified apriori algorithm for finding the association rules among large datasets to promote sales and user interaction. They showed that the proposed algorithm improved the efficiency of generating association rules. Moreover, Davagdorj and Ryu [11] offered an association rule mining method to discover useful patterns, which include medical knowledge, from a medical dataset. They applied the FP growth algorithm to extract a set of association rules. Then, the obtained rules are used to support medical decision-making for interpreting diagnosing patient information. Furthermore, Asadianfam et al. [12] introduced a new approach to improve recommendations that can be used to predict the next navigable page of users. One of the objectives considered in their approach is to provide appropriate recommendations to users who have different profiles from the existing users' profiles. To deal with the objective, the authors used the apriori algorithm to generate association rules from users' behaviors and then made appropriate recommendations. They showed that the generated association rules could increase the overall efficiency of the recommender system. More recently, Miswan et al. [13] proposed a framework of association rule mining in readmission tasks. The proposed framework consisted of two processes, namely, data preprocessing and rule mining extraction. Apriori algorithm is used to extract the hidden input variable patterns and relationships among admitted patients by generating supervised learning rules. The mined rules are discussed and validated by the domain expert, which is a valuable guide in making decisions on targeted patients' clinical resources based on various readmission durations.

Apart from these works, there are also few works exploring the association rule learning using machine learning algorithms. In this context, Hong et al. [14] proposed an agent-based framework for offering personalized services utilizing the extracting users' preferences and association rules. The decision tree algorithm is considered to infer association rules for recommending personalized services for users. Similarly, Zulkernain et al. [15] introduced an intelligent mobile interruption management system. The main idea of their proposed system is to intelligently assist users in their daily activities. To this end, a decision tree algorithm is used to make intelligent decisions. Moreover, Sarker [16] presented an association rule learning approach that can be used to discover a set of nonredundant and useful rules. In their approach, they considered, first, the Naïve Bayes (NB) algorithm to eliminate noise from data and, second, the decision tree algorithm to generate a set of association rules. These algorithms are used to build a robust prediction model that could improve the prediction accuracy. Finally, Basha [17] provided a cardiovascular prediction system that combines the traditional K-nearest neighbor (KNN) algorithm with a GA to extract strong association rules for facilitating the decision-making process. First, the proposed system extracts association rules using the KNN algorithm. Then, the output rules become the population of the GA to remove redundant and irrelevant rules.

Nevertheless, a common weakness that can be found in the majority of discussed works [9–13] mainly stands in the use of traditional association rule mining algorithms, such as FP growth and apriori. This weakness is caused by the narrow applicability of these algorithms due to the huge number of association rules generated [18]. However, these works generate numerous redundant association rules, which lead to generating a huge number of uninteresting rules that become useless in making decisions. This redundant generation makes not only the rule set unnecessarily large but also makes the decision-making process more complex and ineffective. Therefore, the traditional association rule mining algorithms are not able to efficiently extract interesting association rules. Instead, employing machine learning algorithms can overcome this weakness and avoid rule redundancy and conflicts in the mining process. Despite that, in many cases, works based on machine learning algorithms [14–17] could not ensure high accuracy in generating association rules [19]. Furthermore, a limitation in some of these works [9, 13] is that they are only suitable for offline mining and not suitable for dynamic environments where new incoming changes are continuously received at runtime and so decisions may be wrongly predicted based on rules defined a priori. A further important limitation is that certain works like the work of Miswan et al. [13] require domain expert intervention to validate generated association rules. Following the above discussion, we aim to close gaps within the discussed works by proposing an approach that relies on automatically enriching a rule knowledge base with new context-aware rules generated at runtime to perform an efficient decision-making for dynamic environments. The proposed approach focuses on discovering and generating nonredundant and well-performed association rules through the use of machine learning algorithms along with an extended GA to reduce rule redundancy. To address the decision-making accuracy question, the proposed approach represents a hybridization idea that combines the strengths of two supervised machine learning algorithms, the decision tree, and the random tree algorithms. Moreover, it proceeds for a rule transformation to support the automatic enrichment of rule knowledge bases at runtime.

## 3. Proposed Approach

**Figure 1:**
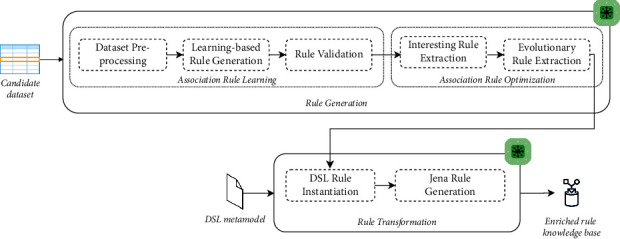
Proposed approach architecture.

In this work, we propose an approach that supports the improvement of decision-making in dynamic environments at runtime by allowing an automatic enrichment of a rule knowledge base with new generated context-aware rules. The proposed approach first aims to generate a concise set of nonredundant association rules following the IF-THEN structure, then to optimize the generated rules, and finally to transform rules to Jena rules for enriching the rule knowledge base and providing appropriate services to users situated in dynamic environments at runtime.  Figure 1 illustrates a schematic view of the proposed approach architecture.

As illustrated in Figure 1, the proposed approach typically consists of two main modules, namely, the rule generation module and the rule transformation module. In the following, we discuss these modules and their roles in generating association rules and transforming them into Jena rules.

### 3.1. Rule Generation Module

The rule generation module is designed to automatically derive association rules needed to meet the changes occurring in users' dynamic environments. That means, it aims to preprocess a candidate dataset, to generate decision trees, and to infer the well-performed association rules in order to further enrich a rule knowledge base and improve the decision-making process at runtime. The present module runs every time when new changes are arrived and a priori rules are deemed not relevant to these changes. This module is defined considering two main phases as shown in Figure 1.

#### 3.1.1. Association Rule Learning

The association rule learning phase is in charge of learning and extracting association rules from a candidate dataset. This phase includes various steps starting from the preprocessing of the dataset to the validation of rules.

*(1) Dataset Preprocessing*. This represents the first step of the association rule learning phase, which is applied to improve the quality of the candidate dataset so as to ensure accurate, consistent, and complete generated decision trees and further association rules. This step is responsible for preparing data from the candidate dataset. Hence, two major stages during the data preprocessing step, namely, data cleaning and data reduction, are performed. The first stage comprises both operations like filling missing data and smoothing noisy data. It aims to replace missing data with the average of existing data, using different traditional imputation methods (i.e., mean and median). To smooth noisy data, we carry out techniques, such as clustering, regression, and binning, to eliminate the noise in the dataset as it rises due to random variation. The second stage is data reduction that is used to obtain a simplified representation of a dataset with relevant data. To do it in the simplest manner, we remove redundant and inconsistent data.

**Figure 2:**
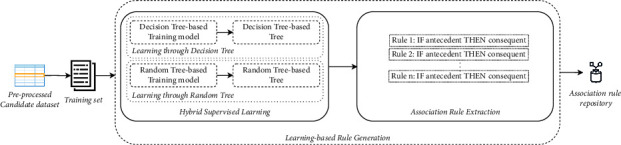
Learning-based rule generation step.

*(2) Learning-Based Rule Generation*. After the data preprocessing step is completed, the learning-based rule generation step takes place first to automatically learn training models for building decision trees, then to derive association rules, and finally to integrate them into the association rule repository. To this end, the present step provides two elementary mechanisms. The main idea of these mechanisms can be simply described in Figure 2.

A first mechanism, called hybrid supervised learning, takes advantage of machine learning and pattern recognition in learning about data, extracting relevant relationships, and eliminating redundancy. Thus, it combines widely adopted supervised machine learning algorithms, namely, J48 decision tree [20] and random tree [21], to train the preprocessed dataset. As far as we know, this is the first time that J48 decision tree and random tree algorithms are combined to the association rule learning. To improve the accuracy of learning, we come up with the idea of hybridizing J48 decision tree and random tree algorithms as they are tree-based techniques that provide the highest accuracy diagram of a decision tree [22]. The resulting training models consist of a set of decisions in a tree structure, which could be utilized to generate rules from each leaf node [23]. As shown in Algorithm 1, this first mechanism trains the J48 decision tree to build the corresponding decision tree on the decision tree-based training model. Subsequently, it applies the random tree algorithm to get the second decision tree on the random tree-based training model.

The second mechanism, called association rule extraction, acquires association rules from previously trained decision trees. To do so, it tracks, in an automated fashion, the path from the root node to each leaf node in both trees in order to detach the set of association rules.

The output of this mechanism is a set of association rules following IF-THEN statements: IF <A> THEN <C>, where the antecedent part <A> represents user's surrounding contextual information such as temporal context, spatial context, social contexts, or others relevant contextual information, for example, “outlook = overcast,” and the consequent part <C> represents their corresponding behavioral activities for decision-making, for example, “play = yes.”

*(3) Rule Validation*. After completing the generation step, the rule validation step is applied through the following methods:

- Rule structure verification, which is responsible for checking whether extracted rules are preserving the inherent IF-THEN structure.
- Rule consistency verification, which is in charge of verifying the consistency of association rules. Given the fact that association rules are made up of antecedent constraints and a consequent constraint, the rule consistency is related to the satisfiability of constraints. To ensure a conflict-free association rule repository, a reasoner is used to enumerate all inconsistent rules, where the consequent constraint does not refer to the antecedent constraint.

#### 3.1.2. Association Rule Optimization

**Figure 3:**
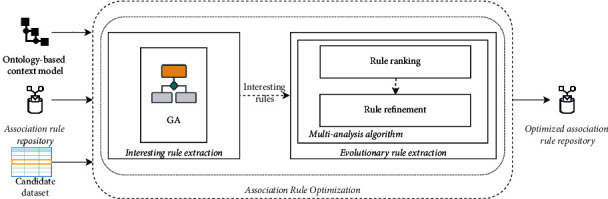
Rule optimization phase.

The association rule optimization is the second and last phase in the rule generation module. The present phase is in charge of identifying the well-performed rules from a set of earlier validated association rules. In this sense, a GA is extended to support a multianalysis technique. The main idea of extending a GA with a multianalysis technique is to optimize the set of association rules to find the well-performed rules by adopting the optimization strategies in GA. To be more precise, there are two main steps in this phase as depicted in Figure 3. First, the interesting rule extraction step is carried out using GA that is a kind of effective optimizing technique owing to the robust and global search ability [24]. Second, the evolutionary rule extraction step involves a multianalysis technique to extend the GA and extract the well-performed association rules.

**Figure 4:**
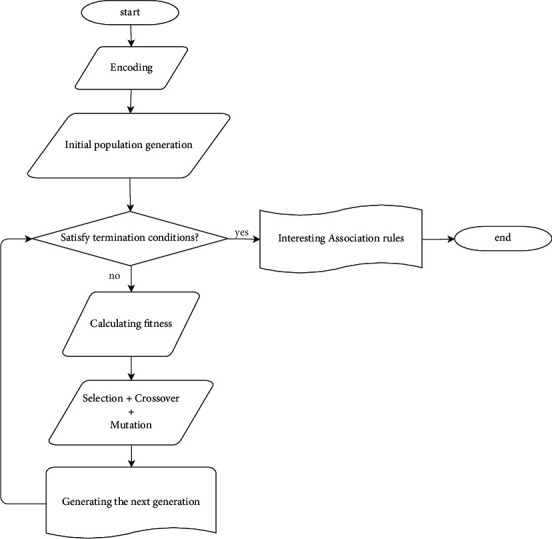
Flowchart of a GA.

*(1) Interesting Rule Extraction*. This step determines the interesting association rules by applying a GA to the candidate dataset. The GA is used to produce a strong level of association rules since supervised learning algorithms may generate irrelevant rules. The motivation behind this choice is twofold: first, the GA is one of the best methods for rule optimization [25], and second, it performs a global search technique to find out interesting rules with less complexity compared to other algorithms [26]. The GA creates an initial population as a collection of chromosomes, in which every chromosome represents an association rule. Then, it evolves the initial population over multiple generations through encoding, selection, crossover, and mutation operations to reach the optimal set of interesting association rules. In the end, it introduces a set of interesting association rules, which satisfies a fitness function. The flowchart of GA is shown in Figure 4.

The specific steps of the GA are as follows:

(i) Step 1. Encoding step: The candidate dataset is encoded to initiate the experimentation of the GA. In our case, a binary encoding schema is used.
(ii) Step 2. Initial population generation: An initial population of size *K* chromosomes is randomly generated as a set of solutions to be optimized. These chromosomes are a representation of the rules generated from the candidate dataset.
(iii) Step 3. Calculating fitness: The fitness value of each chromosome in the population is calculated by a fitness function to find the association rules that their support and confidence are larger than other rules. To this end, a fitness function that has been described in the work of Qodmanan et al. [27] is considered as given in equation.
  (1) $$\begin{array}{c} \text{fitness}=\frac{{\left(1+\sup p\left(A\cup C\right)\right)²}^{2}}{1+\sup p\left(A\right)}. \end{array}$$
In equation (1), supp(A∪C) is the support of the *A* ⟶ *C* and sup*p*(*A*) is the support of the antecedent part of it.
(iv) Step 4. Selection: A chromosome with a high fitness value is selected from the population on the basis of a fitness function.
(v) Step 5. Crossover: The next generation of the population is generated based on the calculated fitness values. The idea behind crossover is to combine the two parent chromosomes to produce two new offspring. The result of crossover is the birth of two new chromosomes. Crossover is carried out according to a defined crossover probability.
(vi) Step 6. Mutation: Mutation randomly changes chosen bits from 0 to 1 or from 1 to 0. It is applied to the new offspring with a certain mutation probability. The purpose is to maintain diversity among the different generations to increase the global optimization of the GA.
(vii) Step 7: After a series of selection, crossover, and mutation, the GA is stopped when the generated chromosomes meet the optimality or the maximum number of generations. Otherwise, it turns back to step 3 to continue the rule optimization.

*(2) Evolutionary Rule Extraction*. In the previous step, the interesting association rules are selected. However, we cannot get the well-performed rules that could lead to achieve the appropriate decision-making performance due to the fact that the fitness function, defined in GA, might be in conflict. To deal with this issue, the evolutionary association rule step proposes a multianalysis technique for extending a GA in order to enhance the rule optimization result. For that, rule ranking and refinement processes are applied as shown in Figure 3. The multianalysis technique starts with the rule-ranking process that is in charge of automatically analyzing and ranking the interesting association rules and the generated rules from supervised learning algorithms regarding their frequency of occurrence and their statistical information. In the proposed rule-ranking process, the rule occurrence frequency is considered as the highest priority to classify rules, followed by the statistical information, such as the fitness function weight. Then, the rule refinement process is performed to derive the set of well-performed rules. This process begins with finding a user who is related to generated rules, loading the user profile of the corresponding user from the ontology-based context model and inferring the well-performed rules that could significantly match with the profile.

### 3.2. Rule Transformation Module

**Figure 5:**
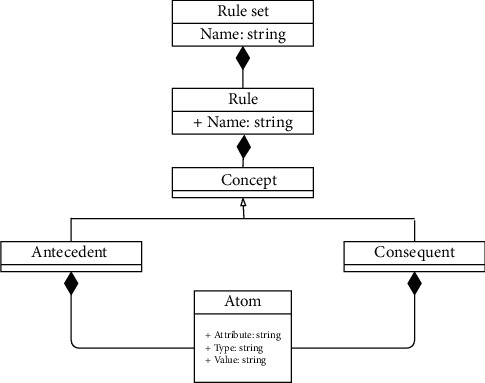
Jena rule definition metamodel.

**Figure 6:**
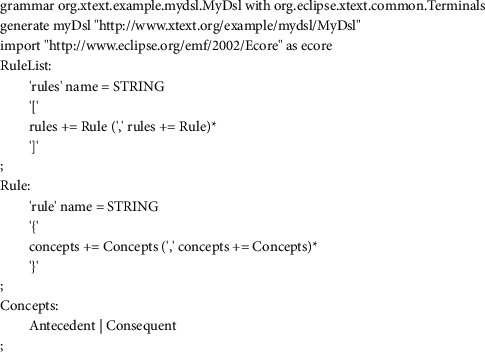
A fragment of the textual description of the DSL metamodel.

The second module in the proposed approach is the rule transformation module since generating IF-THEN association rules is not enough. This module aims to obtain Jena rules from the well-performed association rules in order to reason over an ontology-based context model and to infer high-level knowledge using the Jena inference engine. The motivation to opt for Jena is its built-in support for rule-based inference over RDF and OWL [28]. In this sense, the rule transformation keeps track of the well-performed association rule set, generated in the previous module, and transforms them into Jena rules according to the rule syntax of Jena. In the following, rules in Jena format induced from the well-performed association rules are loaded by the module into the rule knowledge base for an automatic enrichment purpose at runtime. The rule transformation module targets design time and runtime. At design time, a rule translation is ensured, where a metamodel for Jena rules is proposed to specify the abstract syntax of a Jena rule.  Figure 5 shows the metamodel for Jena rules. A rule set consists of rules. A Jena rule has a name and is composed of concepts. A concept is interpreted as an antecedent and as consequent. Therefore, a Jena rule can contain one or more atoms in the antecedent part and only one atom in the consequent part. Each atom has an attribute, type, and value. Then, the proposed metamodel is translated into a domain-specific language (DSL) metamodel. The latter is defined as a textual structure using Xtext. A fragment of the DSL metamodel is depicted in Figure 6.

During runtime, the transformation module performs two paramount phases: (i) instantiation of the DSL metamodel and (ii) generation of the Jena rules on the basis of the DSL model as depicted in Figure 1.

#### 3.2.1. DSL Rule Instantiation

The DSL rule instantiation phase is dedicated to introduce a DSL model, which is a formal rule specification of the defined DSL metamodel. The DSL model represents the well-performed obtained association rules. Here, the rule instantiation is automatically performed at runtime.

#### 3.2.2. Jena Rule Generation

The Jena rule generation phase is considered for automatically generating the corresponding Jena rules based on the DSL model using Xtend as a transformation language. Therefore, a set of Jena rules is generated from the DSL model while the semantic of the DSL metamodel is well-defined.

## 4. Experimental Results

In this experimental evaluation, we provide an example of generated rules by our proposed approach. In addition, we discuss the effectiveness of our proposed approach. For this, a range of experiments was carried out to investigate the effectiveness in terms of the number of rules, performance, and computational time.

### 4.1. Experimental Setup

All experiments were conducted on six benchmark datasets of varying complexity. The name, number of instances, number of attributes, and number of class labels in each dataset acquired from the UCI Machine Learning Repository are included in Table 1.

For evaluation purposes, we utilized standard open-source implementations of supervised learning algorithms and association rule mining algorithms provided by Weka [29] in a 10-fold cross-validation evaluation protocol in order to get accurate results for all datasets. In the 10-fold cross-validation protocol, the entire benchmark datasets are partitioned into ten parts of equal size, nine parts of them are used at a time for training, and the remaining one is used for testing. The process is repeated ten times, with different partitions used as training data and test data. In addition, we set the minimum expected weighted confidence threshold to 1 and support threshold to 0.5 in order to generate the results described in the next experiments. Furthermore, the various GA parameters were selected. Crossover and mutation probabilities were taken, respectively, as 0.5 and 0.01. The size of the initial population depends on the number of the generated rules from the benchmark datasets. Thus, the initial population size ranges from 14 to 439 and the maximum number of generations is set to 100.

### 4.2. Experimental Metrics

For analyzing the effectiveness of the proposed approach, some well-known metrics were used. Therefore, the following parameters were considered for these metrics:

- True positive (TP) is the number of rules positively predicted that is actually positive.
- True negative (TN) is the number of rules negatively predicted that is actually negative.
- False positive (FP) is the number of rules positively predicted that is actually negative.
- False negative (FN) is the number of rules negatively predicted that is actually positive.

Based on the previous parameters, the following metrics were proposed for the evaluation:

(i) Precision reflects the ability of an approach to return relevant rules among a set of irrelevant and relevant rules. The precision can be computed by equation.
  (2) $$\begin{array}{c} \text{Precision}=\frac{TP}{TP+FP}. \end{array}$$
(ii) Recall reflects the ability of an approach to return relevant rules only. The recall is defined as given in equation.
  (3) $$\begin{array}{c} \text{Recall}=\frac{TP}{TP+FN}. \end{array}$$
(iii) Accuracy reflects the ability of an approach to return the accurate rules over the all rules made in the dataset as can be seen in equation.

(4) $$\begin{array}{c} \text{Accuracy}=\frac{TP+TN}{TP+FP+TN+FN}. \end{array}$$

Apart from these metrics, we considered the computational time that reflects the average time required for generating the set of IF-THEN association rules in terms of second.

### 4.3. Rules for Knowledge Base Enrichment

We present an example of context-aware rules generated from the weather dataset mentioned above using our approach. The candidate dataset contains four condition attributes, such as outlook, temperature, humidity, windy, and one decision attribute, that is, “play.” It is considered to generate rules that could help in making decisions regarding whether a user could go outside for playing or not. First, we show, in Table 2, a sample of generated IF-THEN rules obtained through training both decision tree and random tree algorithms. Looking more closely at Table 2, we can notice that the antecedent part of the rules reflects users' contextual knowledge, and the consequent part represents their associated behavioral actions. Hence, real-time users' environmental contexts such as outlook, wind, humidity, or temperature are used to make the play decision.

Then, we illustrate, in Tables 3 and 4, a sample of well-performed rules extracted from the generated rules through the optimization process considered in the proposed approach. As illustrated in Tables 3 and 4, the well-performed discovered association rules vary from one user to another according to their current profile. For example, a user with a pollen allergy, on a windy day, cannot go outside since the wind can trigger pollen allergy symptoms most. As a result, association rules, such as R9 and R10, which stated a day is windy (windy = true) and a user could have fun playing outside (play = yes), are overlooked for the user with a pollen allergy.

Finally, after the automatic DSL metamodel instantiation and transformation, we list, in Tables 5 and 6, a sample of the well-performed rules transformed to Jena rules to enrich the rule knowledge base and improve the decision-making process at runtime. Therefore, we can conclude that our proposed approach is capable of generating rules that could enrich a rule knowledge base.

### 4.4. Effectiveness Analysis

#### 4.4.1. Rule Analysis

**Figure 7:**
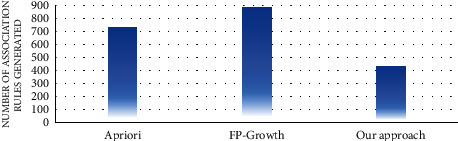
Rule analysis for the breast dataset.

**Figure 8:**
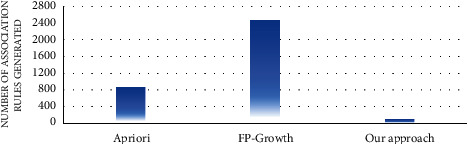
Rule analysis for the heart dataset.

**Figure 9:**
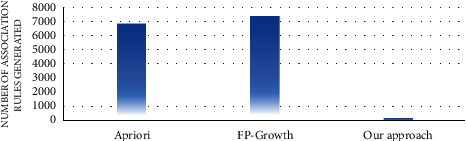
Rule analysis for the hepatitis dataset.

**Figure 10:**
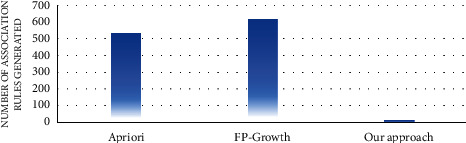
Rule analysis for the weather dataset.

**Figure 11:**
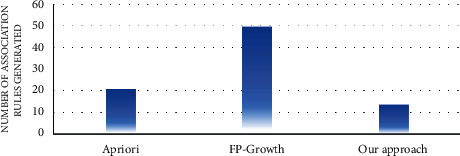
Rule analysis for the iris dataset.

**Figure 12:**
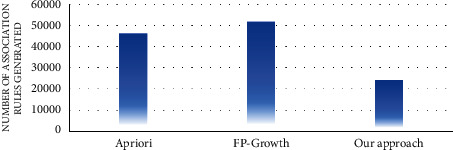
Rule analysis for the adult dataset.

In the first experiment, the number of association rules generated by the proposed approach and the traditional algorithms of association rule mining, such as apriori and FP growth, was evaluated. Figures 7–12 illustrate the number of generated rules for the different candidate datasets.

Observing Figures 7–12, we can see that the traditional algorithms generate the highest number of rules, while our approach generates the lowest number of rules on all candidate datasets. In particular, our approach generates 4,103 association rules on average against 6 datasets, whereas the apriori and FP growth algorithms derive 9,211 and 10,582 on average, respectively. Thus, the results show that the number of generated association rules using traditional algorithms is large and huge. The reason beyond these results is that traditional algorithms simply take into account all combinations of attributes while generating rules. In contrast, the results indicate that the number of generated rules by our approach is small. The downtrend reveals that our approach could generate the minimum number of association rules comparing the apriori and FP growth and could keep the number of discovered rules as small as possible. As a result, for a high confidence value, traditional algorithms satisfy significantly more associations than our proposed approach. Therefore, our proposed approach could generate a reasonably smaller number of association rules compared with traditional association rule mining algorithms since it abandons the redundant rules and retains the nonredundant rules.

#### 4.4.2. Performance Analysis

**Figure 13:**
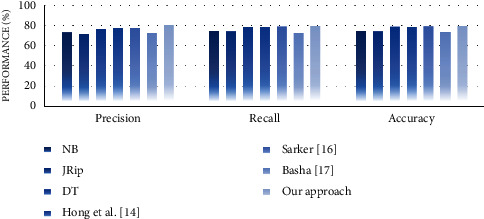
Performance analysis for the breast dataset.

**Figure 14:**
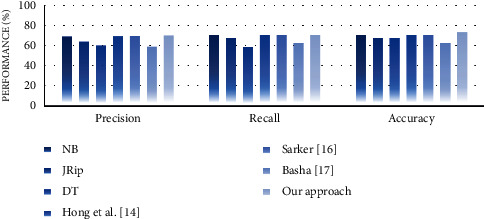
Performance analysis for the heart dataset.

**Figure 15:**
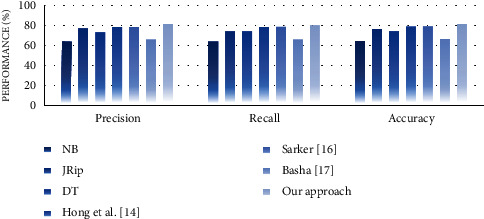
Performance analysis for the hepatitis dataset.

**Figure 16:**
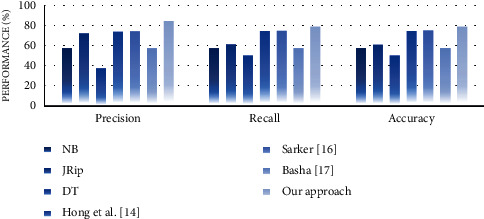
Performance analysis for the weather dataset.

**Figure 17:**
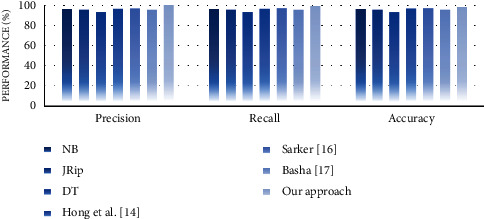
Performance analysis for the iris dataset.

**Figure 18:**
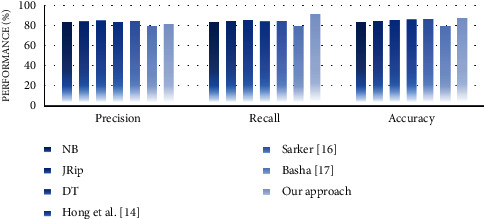
Performance analysis for the adult dataset.

In the second experiment, we discussed the effectiveness of our approach in terms of performance measures such as precision, recall, and accuracy. For this purpose, we compare the performance of the proposed approach with well-known supervised learning algorithms, namely, NB, JRip, and decision table (DT). The reason for selecting these algorithms is that they generate rule-based classifiers and have high performance compared with other algorithms [30]. We also compare the performance of our approach with some similar state-of-the-art works, including Hong et al. [14], Sarker [16], and Basha [17]. Experimental results on performance measures are highlighted in Figures 13–18.

Our observations on candidate datasets show that our approach consistently outperforms the compared supervised learning algorithms for generating association rules by maximizing the precision and recall. In addition, our observations reveal that our proposed approach achieved better accuracy than NB, JRip, and DT on heart, hepatitis, weather, and iris datasets. For instance, the accuracies for NB, JRip, and DT on the iris dataset are 95.33%, 96%, 95.3%, and 92.66%, respectively, whereas for the proposed approach the accuracy is 98%. Moreover, the obtained results confirm that our approach has outstanding performance compared with state-of-the-art works. For instance, we achieve an approximately 2% and 3.2% accuracy gain compared with the works of Hong et al. [14], Sarker [16], and Basha [17] when dealing with breast and adult datasets, respectively. Thus, obtained results proved that our proposed approach tends to get reasonably high accuracy on all datasets. Therefore, we can conclude that our proposed approach is more effective relative to the compared supervised learning algorithms and state-of-the-art works while generating association rules since we capture association rules from both more performant supervised learning algorithms that lead to improve the performance results.

#### 4.4.3. Computational Time Analysis

In the third experiment, we compared and analyzed the computational time of the proposed approach, the supervised learning algorithms mentioned earlier, and the state-of-the-art works [14, 16]. In this experiment, we did not consider the work presented by Basha [17] since its GA is not publicly available. To this end, the sizes of the datasets were fixed since the computational time may vary based on the dataset size.  Table 7 illustrates the time consumed by the proposed approach for generating association rules against the selected supervised learning algorithms and the state-of-the-art works in all datasets.

From the illustrated results, the average time spent on each dataset to generate a set of association rules is 0.38 seconds in the proposed approach. The experimental results clearly show that the computational time of the proposed approach is slightly increased compared with the selected supervised learning algorithms and with the state-of-the-art works, but the computational time on the whole is not much different. The slight increase can be explained by the fact that we apply the GA for optimization on the basis of both supervised learning algorithms, which could slightly increase the time complexity of the rule generation process compared with the selected supervised learning algorithms.

## 5. Discussion

We discuss the encouraging obtained results of the proposed approach. First, our approach outperformed compared to the well-known traditional association rule mining algorithms in terms of the number of rules by eliminating the redundant generation for each dataset. It generated, on average far, fewer association rules than those generated by the traditional algorithms included in the comparison. Thus, it provided a reasonably smaller number of rules on smaller and bigger datasets compared to the traditional algorithms. This is due to the fact that our approach is based on hybrid supervised learning that takes advantage of machine learning to extract the relevant relationships and to eliminate the redundancy while generating association rules. In brief, our approach significantly reduces the total number of generated rules and outputs a well-performed set of association rules using the GA. Such nonredundant and well-performed association rule generation makes our approach more effective and can be used to automatically improve a decision-making process regarding changes occurring in users' surrounding environments at runtime. Second, our approach exceeded the well-known selected supervised learning algorithms and certain state-of-the-art works in terms of precision, recall, and accuracy. In particular, our approach achieved the best accuracy on heart, hepatitis, weather, and iris datasets among all selected supervised learning algorithms. However, it got slightly worse results in terms of accuracy on breast and adult datasets since imbalanced datasets may lead to slightly worse accuracy results. Even though not achieving the best accuracy results on these datasets, our approach achieves almost reasonably high accuracy in most datasets. Moreover, we must stress that the main advantage of our approach in contrast to the compared state-of-art works is its ability to achieve a good trade-off between precision, recall, and accuracy on multiple datasets. Third, our approach could be somewhat more time-consuming than the selected supervised learning algorithms and the compared state-of-the-art works. The slight increase in time is reasonable due to the hybridization and optimization. However, this loss of the little running efficiency will result in a significant improvement in the quality of the results in terms of the number of rules, precision, recall, and accuracy. Regarding the time-consuming results, it is noted that the effectiveness of the proposed approach does have room for improvement, which is also the need for further improvement in the future work.

Overall, the findings of the experimental study reveal that our approach can (i) effectively minimize the issues of redundant rule generation, (ii) provide a promising performance and a high accuracy to extract a concise set of association rules, and (iii) take slightly more time for generating a set of well-performed rules.

## 6. Conclusions

This work aimed to propose an approach for the decision-making improvement to face dynamic environments at runtime, where the enrichment of a rule knowledge base is performed through automatically generating and transforming context-aware rules. The proposed approach focused first on generating nonredundant association rules with a hybrid supervised learning mechanism, second on optimizing association rules using an extended GA, and finally on transforming association rules to context-aware rules in Jena syntax. As part of this work, we presented an experimental evaluation to assess the effectiveness of the proposed approach. The results showed that the proposed approach has the potential for achieving the best results in terms of the number of rules, precision, recall, and accuracy among all compared algorithms. Moreover, these results pinpointed the limitation of the proposed approach in terms of time-consuming that is mainly due to hybridization and optimization. Furthermore, in the near future, we intend to apply our approach to real scenarios for a context-aware system.

## Figures and Tables

**Algorithm 1:**
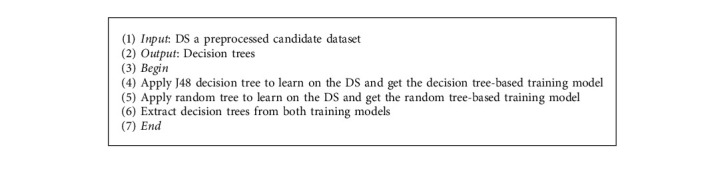
Hybrid with decision tree and random tree.

**Table 1:** Characteristics of datasets used in experiments.

Dataset	Number of instances	Number of attributes	Number of classes
Breast	286	10	2
Heart	270	13	4
Hepatitis	155	19	2
Weather	14	5	3
Iris	150	4	3
Adult	48842	15	2

**Table 2:** A sample of generated rules from the weather dataset.

Rules	IF-THEN rules
R1	IF outlook = overcast THEN play = yes
R2	IF outlook = rainy AND windy = false THEN play = yes
R3	IF outlook = rainy AND windy = true THEN play = no
R4	IF outlook = sunny AND humidity = high THEN play = no
R5	IF outlook = sunny AND humidity = normal THEN play = yes
R6	IF humidity = normal AND windy = false THEN play = yes
R7	IF humidity = high AND outlook = rainy AND windy = false THEN play = yes
R8	IF humidity = high AND outlook = rainy AND windy = true THEN play = no
R9	IF humidity = normal AND windy = true AND temperature = hot THEN play = yes
R10	IF humidity = normal AND windy = true AND temperature = cool AND outlook = overcast THEN play = yes

**Table 3:** A sample of well-performed rules for a user with no allergy history.

Rules	IF-THEN rule	Occurrence frequency	Fitness value
R1.1	IF outlook = overcast THEN play = yes	1	0.743
R1.2	IF humidity = normal AND windy = false THEN play = yes	1	0.686
R1.3	IF humidity = normal AND windy = true AND temperature = cool AND outlook = overcast THEN play = yes	1	0.675
R1.4	IF humidity = normal AND windy = true AND temperature = hot THEN play = yes	1	0.611

**Table 4:** A sample of well-performed rules for a user with a pollen allergy.

Rules	IF-THEN rule	Occurrence frequency	Fitness value
R2.1	IF outlook = overcast THEN play = yes	1	0.743
R2.2	IF humidity = normal AND windy = false THEN play = yes	1	0.686
R2.3	IF outlook = sunny AND humidity = high THEN play = no	2	0.611
R2.4	IF outlook = rainy AND windy = true THEN play = no	1	0.611

**Table 5:** A sample of well-performed rules in Jena format for a user with no allergy history.

Rules	Jena rules
R1.1	(rule R1: (?outlookValue uni:outlook “overcast”) ⟶ (?playValue uni:play “yes”))
R1.2	(rule R2: (?humidityValue uni:humidity “normal”) (?windyValue uni:windy “false”) ⟶ (?playValue uni:play “yes”))
R1.3	(rule R3:(?humidityValue uni:humidity “normal”) (?windyValue uni:windy “true”) (?temperatureValue uni:temperature “cool”) (?outlookValue uni:outlook “overcast”) ⟶ (?playValue uni:play “yes”))
R1.4	(rule R4: (?humidityValue uni:humidity “normal”) (?windyValue uni:windy “true”) (?temperatureValue uni:temperature “hot”) ⟶ (?playValue uni:play “yes”))

**Table 6:** A sample of well-performed rules in Jena format for a user with a pollen allergy.

Rules	Jena rules
R2.1	(rule R1: (?outlookValue uni:outlook “overcast”) ⟶ (?playValue uni:play “yes”))
R2.2	(rule R2: (?humidityValue uni:humidity “normal”) (?windyValue uni:windy “false”) ⟶ (?playValue uni:play “yes”))
R2.3	(rule R3: (?outlookValue uni:outlook “sunny”) (?humidityValue uni:humidity “high”) ⟶ (?playValue uni:play “no”))
R2.4	(rule R4: (?outlookValue uni:outlook “rainy”) (?windyValue uni:windy “true”) ⟶ (?playValue uni:play “no”))

**Table 7:** Computational time analysis for our six datasets in seconds.

Dataset	NB	JRip	DT	Hong et al. [14]	Sarker [16]	Our approach
Breast	0.05	0.04	0.02	0.03	0.05	0.06
Heart	0.02	0.05	0.06	0.04	0.06	0.10
Hepatitis	0.05	0.04	0.03	0.03	0.05	0.08
Weather	0.02	0.02	0.05	0.04	0.06	0.16
Iris	0.02	0.01	0.02	0.02	0.04	0.06
Adult	1.13	1.48	1.94	1.08	1.10	1.82
Average	0.29	0.27	0.35	0.21	0.23	0.38

## Data Availability

The public datasets were collected from the UCI Machine Learning Repository. Requests for dataset access should be made to https://archive.ics.uci.edu/ml/index.php/.
